# Anxiety and Depression in Chinese Students During the COVID-19 Pandemic: A Meta-Analysis

**DOI:** 10.3389/fpubh.2021.697642

**Published:** 2021-08-17

**Authors:** Yaoyao Zhang, Xiuqin Bao, Jiaxin Yan, Hualing Miao, Cheng Guo

**Affiliations:** ^1^Department of Psychology, Southwest University, Chongqing, China; ^2^Department of Medcine, Yan'an University, Yan'an, China

**Keywords:** COVID-19, anxiety, depression, China, meta-analysis

## Abstract

**Background:** The novel 2019 coronavirus disease (COVID-19) pandemic has spread rapidly worldwide and poses a global health threat.

**Aims:** This study assessed the prevalence of anxiety and depression symptoms in Chinese students during the COVID-19 pandemic and explored potential moderating factors.

**Methods:** We searched English and Chinese databases using pertinent keywords for articles published and unpublished, up until November 2020. The estimate of the overall prevalence of anxiety and depression was conducted through a random-effects model.

**Results:** A total of 31 cross-sectional studies were included. The overall prevalence of anxiety and depression symptoms in Chinese students during the COVID-19 pandemic was 24.0% (95% CI [20.0–29.0%]) and 22.0% (95% CI [18.0–27.0%]) respectively. Subgroup analyses revealed that Chinese middle school students were at heightened risk of anxiety, while university students were at heightened risk of depression. Students who lived in higher-risk areas presented severe anxiety and depression, especially during the late period of the COVID-19 epidemic.

**Conclusions:** Overall, during the COVID-19 pandemic, there was a high prevalence of anxiety in Chinese students and a high prevalence of depression among Chinese students in high-risk areas. Therefore, comprehensive and targeted psychological interventions should be developed to address the mental health of students in different grades, especially in high-risk areas and during the late period of the COVID-19 pandemic.

## Introduction

The novel 2019 coronavirus disease (COVID-19)—caused by SARS-CoV-2—is an emerging, rapidly evolving pandemic (1). The first case of acute infectious pneumonia caused by COVID-19 emerged from Wuhan, China (2, 3). Due to the high infectiousness of COVID-19 and its consequent wide and rapid spread, Chinese schools and factories closed, and the government implemented home isolation (4). Furthermore, the impact of the COVID-19 outbreak on mental health remains poorly understood, although many Chinese people have exhibited a tendency toward increased mental health issues and sensitivity to social risks within China (5, 6).

Anxiety and depressive symptoms have been common mental health problems for populations during the COVID-19 pandemic (2, 7). Students, as a vulnerable population, are relatively prone to anxiety and depression symptoms (8), and the COVID-19 pandemic has led to short- and long-term anxiety and depression among students (9, 10). Prolonged anxiety and depression are associated with increased levels of negative mental health of students, resulting in symptoms such as fear, stress, insomnia (10, 11), and behaviors such as aggression, smartphone addiction, and suicide (12). However, the proportion of students who experienced anxiety and depression during the COVID-19 is unclear. China has experienced a relatively complete outbreak process because it took a series of measures to control the outbreak as early as possible. Therefore, it is necessary to explore the incidence of anxiety and depression symptoms of students in China during the COVID-19 to provide data that may help in controlling the global COVID-19 pandemic.

Existing systematic analyses and meta-analyses have assessed the prevalence of anxiety and depression among children and adolescents during the COVID-19 pandemic. The systematic analysis of Nearchou et al. (13), which included adolescents ≤18 years old, found that COVID-19 increased adolescent depression and anxiety. The meta-analysis of Panda et al. (14) revealed that the overall prevalence of anxiety and depression among children worldwide was 34.5 and 41.7%, respectively. Luo et al. (15) indicated that the pooled prevalence of depressive symptoms in Chinese university students was 26.0% during the COVID-19 pandemic. However, to the best of our knowledge, no meta-analysis has evaluated the prevalence of anxiety and depression among Chinese students overall. The results of extant studies of the level of anxiety and depression in Chinese students during the COVID-19 pandemic are inconsistent. Some studies reported prevalence of anxiety of 24.9% (16) and depression of 16.5% (17), whereas other studies reported a 37.4% prevalence of anxiety and 43.7% prevalence of depression in students (18). In addition, little is known about the effect of potential factors that may influence the overall prevalence of anxiety and depression of students during the COVID-19 pandemic.

Based on risk theory and the spatial relationship of population outflow, two factors—pandemic risk areas and pandemic development progression—may be related to the prevalence of anxiety and depression (19, 20). Furthermore, the substantial levels of anxiety and depression caused by COVID-19 and their severity may also be associated with increased age (21, 22). These three potential factors (pandemic risk areas, pandemic period, and study grade) may have moderating effects on the prevalence of anxiety and depression of students (23).

This meta-analysis provides a timely assessment of the prevalence of anxiety and depression among students in China during the early period of the COVID-19 crisis. We further explored how different pandemic risk areas, pandemic development processes, and study grades affected students' anxiety and depression symptoms to inform recommendations for the prevention of, and interventions against, anxiety and depression during the COVID-19 pandemic.

## Methods

### Search Strategy

This study was performed according to PRISMA. Two authors (the first and second author) independently searched the English databases Web of Science, PubMed, Medline, Embase, PsycInfo, and Google Scholar; and the Chinese databases Wanfang, China National Knowledge Infrastructure, and China Science and Technology Journal. Subsequently, we manually searched the references of selected studies, up to November 2020. A third person participated in the discussion if there were discrepancies. Appropriate keywords were used to search, including (*2019-ncov* OR *coronavirus* OR *corona virus* OR *novel coronavirus pneumonia* OR *COVID-19*), (*depression* OR *depressive* OR *Depression*), (*anxiety* OR *mental health problem*), (*children* OR *adolescents* OR *student* OR *youth*), (*China* OR *Chinese*).

### Inclusion and Exclusion Criteria

Studies that met the following criteria were included: (a) Participants were Chinese primary, secondary, or undergraduate students; however, we excluded students with severe psychological distress or posttraumatic stress disorder. (b) The outcome was the prevalence of anxiety and depression among Chinese students during the COVID-19 pandemic. However, studies that referred to other mental health problems (e.g., stress, dementia) or behavioral problems (e.g., suicide, insomnia) were excluded. Although stress and anxiety are often used interchangeably (24), most researchers agree that the definitions of stress and anxiety are different (25, 26). Stress is an emotional and physical tension in response to threat, while anxiety is the body's natural response to stress (27, 28). Based on the different definitions of stress and anxiety, stress was excluded from this study. (c) Study design included cross-sectional studies (field or online surveys). We excluded review research or research plans with incomplete or unidentified data (29), conference abstracts or case reports, studies with incomplete data, and research in duplicate publications.

### Quality Evaluation

The Agency for Healthcare Research and Quality (AHRQ) was specifically designed to evaluate the quality of cross-sectional studies in systematic reviews (30). The AHRQ includes 11 items that are answered as “yes,” “no,” or “unclear.” When the quality assessments of the two authors differed, the original articles were re-examined by a third person until a quality rating was agreed upon.

### Data Extraction and Code

We developed a data extraction table. The extracted contents included the author(s), year, time point of the pandemic, pandemic area, measurement scale, the method of completing the scale, age of the participants, total sample size, number of persons with anxiety or depression, and the prevalence of anxiety and depression. In addition, two researchers independently extracted and coded the data. When there was a discrepancy, discussions were conducted with a third person to reach a final conclusion.

The pandemic area was divided into three levels of risk (31): (1) Wuhan and Hubei provinces were coded as higher-risk areas, (2) the cities around Hubei province but not Hubei (e.g., Chongqing, Henan) were coded as medium-risk areas, (3) large central cities with a large floating population (e.g., Beijing, Shanghai, Guangdong) were coded as lower-risk areas, and (4) areas far away from high-risk areas of the pandemic and large central cities (e.g., the province of Xinjiang, Qinghai, Ningxia) were coded as low-risk areas.

The COVID-19 pandemic development in China was coded into four stages based on Baidu migration big data and geographic information technology (31): (1) occurrence and recessive spread (from December 2019), (2) rapid spread and outbreak (January 2020), (3) diffusion containment (February 2020), (4) and diffusion attenuation (after March 2020). The study participants were primary school students, middle school students, and university students.

### Statistical Analyses

We used a random effects model implemented in R software (Version 3.5.1) (32) to combine the prevalence of anxiety and depression. The results were displayed using a forest plot. Moreover, we used a funnel plot and sensitivity tests to identify publication bias from a qualitative and quantitative perspective. When Egger's linear regression test was non-significant (*p* > 0.05), publication bias was not considered a concern. Trim-and-fill was utilized to examine the publication bias when Egger's linear regression test was significant (*p* < 0.05) (33).

Heterogeneity was assessed through *I*^2^ tests and *p*. *I*^2^ statistics assessed the magnitude of heterogeneity (34). We considered that there was no obvious heterogeneity if *I*^2^ < 50% and *p* > 0.1, while there was heterogeneity if *I*^2^ > 50% and *p* < 0.1. To consider potential moderating factors that may have affected heterogeneity, we conducted subgroup analyses of the pandemic development processes, pandemic area, study grades, and measurement evaluation tools.

## Results

### Selection of Studies

**Figure 1:**
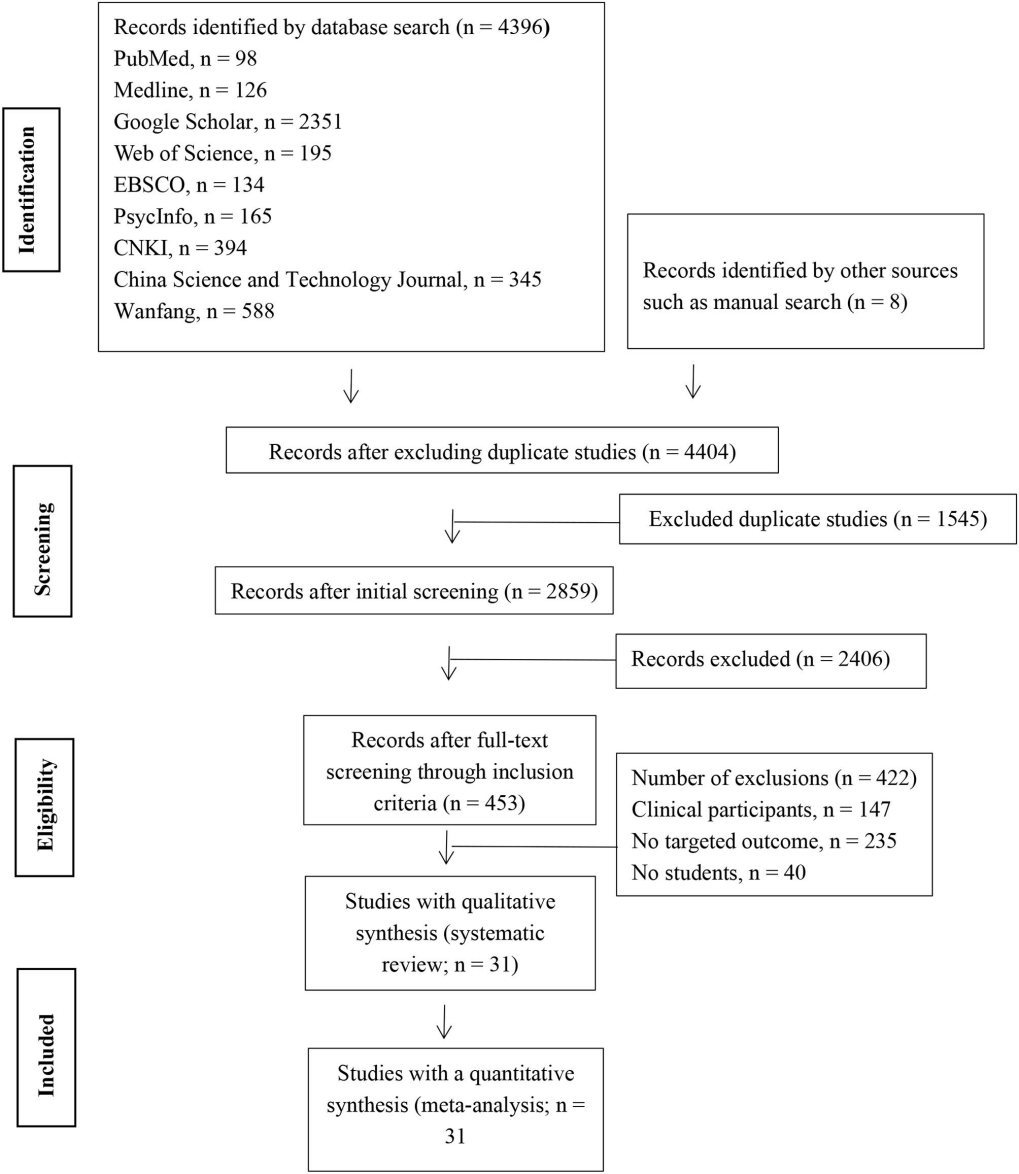
PRISMA literature screening process.

Initially, 4,396 studies were identified on this topic through nine electronic databases and eight studies through manual searches. Subsequently, we removed 1,545 duplicates and 2,821 studies that did not meet the inclusion criteria of this review. Finally, a total of 31 studies were included in this meta-analysis. The flow process is shown in Figure 1.

### Study Characteristics

Study characteristics are displayed in Appendix A. Thirty-one cross-sectional studies were included: 30 studies of anxiety (*n* = 203,678) and 28 of depression (*n* = 78,330). The sample sizes ranged from 84 to 70,158 for anxiety and 84 to 17,420 for depression. Studies were conducted from January to March 2020. However, six studies of anxiety and eight studies of depression did not report measuring time. Regarding risk area, the number of studies that covered higher-risk, medium-risk, lower-risk, and low-risk areas were three, 10, seven, and 16, respectively. Moreover, 25 studies involved the pandemic period of occurrence and recessive spread, seven involved the pandemic period of rapid spread and outbreak, five involved the pandemic period of diffusion containment, and two involved the pandemic period of diffusion attenuation. All studies were conducted using online self-completed questionnaires. Most used reliable and valid assessment tools to measure anxiety and depression symptoms. The tools used to measure anxiety included the Self-rating Depression Scale (SDS), the 9-item Patient Health Questionnaire (PHQ-9), the 7-item measure of Generalized Anxiety Disorder (GAD-7), a self-designed questionnaire, the Screen for Child Anxiety Related Emotional Disorders (SCARED), and the Self-Rating Anxiety Scale (SAS). The tools used to measure depression included a self-designed questionnaire, the Self-report Inventory (SCL-90), the Psychological Questionnaires for Emergent Events of Public Health (PQEEPH), and the Children's Depression Inventory (CDI); see Table 1.

**Table 1:** Summary of the characteristics of the included studies.

**References**	**Anxiety**		**Depressive**		**Participant**	**Outcome**	**City**	**Age (years)**	**Source**	**Time**	**Data**	**Measure**
	**Case**	***n***	**Case**	***n***								
Zhao (35)	34	376			Undergraduate	A	N	N	OF	N	N	SMQ > 50
Wang and Zhao (36)	557	3,611			Undergraduate	A	10 provinces	18–24	OF	DC	February	Self-design
Mo et al. (37)	931	4,928			Elementary student	A	Anhui	7–16	OF	DA	March	SCARED ≥ 23
Mo et al. (37)	114	464			Middle student	A	Anhui	7–16	OF	DA	March	SCARED ≥ 23
Lin and Liu (38)	3,986	10,336	3,464	10,336	Middle student	A D	Sichuan, Chongqing, Shandong	N	OF	DA	2.7–3.20	Self-design
Chang et al. (39)	900	3,881	659	3,881	Undergraduate	A D	Guangdong	N	N	DC	1.31–2.3	GAD-7 ≥ 6 PHQ-9 ≥ 5
Xiao et al. (40)			146	471	Undergraduate	D	Wuhan	N	OF	DA	February	PHQ-9 ≥ 5
Xiao et al. (40)			539	2,082	Undergraduate	D	Hubei Province	N	OF	DA	February	PHQ-9 ≥ 5
Xiao et al. (40)			88	302	Undergraduate	D	Around Hubei Province	N	OF	DA	February	PHQ-9 ≥ 5
Xiao et al. (40)			302	1,111	Undergraduate	D	Other provinces	N	OF	DA	February	PHQ-9 ≥ 5
Cai et al. (17)			1,672	17,420	Undergraduate	D	Guangdong	20.1 ± 2.0	OF	DA	1.31–2.4	PHQ-9 ≥ 5
Fan et al. (41)	2,066	4,148			Undergraduate	A	11 provinces	N	OF	DA	2.23–2	SAS ≥ 50
Sun et al. (42)	998	1,682	998	1,682	Undergraduate	A D	Shandong	N	OF	N	N	Self-design
Ma (43)	118	516	138	516	Undergraduate	A D	Taiyuan	N	OF	DC	February	SCL-90 > 39
Tang and Ying (44)	1,047	3,512	924	3,512	Middle student	A D	Sichuan	N	OF	N	N	SAS ≥ 50
Zhang et al. (45)	1,829	7,833			Undergraduate	A	multicity	N	OF	DA	2.4–2.7	GAD-7 ≥ 5 PHQ-9 ≥ 5
He et al. (46)	1,047	2,895	1,410	2,895	Middle student	A D	N	N	OF	DA	2.20–10	PQEEPH ≥ 1
Ding and Hu (47)	1,039	3,055	303	3,055	Undergraduate	A D	Fujian	N	OF	DC	January	Self-design
Wang and Xu (48)	197	410			Middle student	A	33 provinces		OF	OF	March	GAD-7 ≥ 5
Yu et al. (49)	13	2,074	53	2,074	Middle student	A D	Fujian	N	OF	DA	2.9–10	Self-design
Tang et al. (50)	19	640	19	640	Elementary student	A D	N	N	OF	DA	February	SAS ≥ 50 CDI ≥ 19
Tang et al. (50)	46	233	46	233	Middle student	A D	N	N	OF	DA	February	SAS ≥ 50 CDI ≥ 19
Zhou et al. (18)	3,020	8,079	3,533	8,079	Middle student	A D	21 provinces	12–18	OF	OF	3.8–15	GAD-7 ≥ 5 PHQ-9 ≥ 5
Li et al. (51)	87	396			Elementary student	A	Shanxi	8–18	OF	RO	N	SCARED ≥ 25
Liu et al. (52)	86	611	101	611	Undergraduate	A D	Beijing	7–23	OF	N	N	SAS ≥ 50 SDS ≥ 53
Cao et al. (16)	1,778	7,143			Undergraduate	A	Shanxi	N	N	N	N	GAD-7 ≥ 5
Cao et al. (53)	18,568	56,064			Elementary student	A	Chengdu	N	OF	DA	2.6–10	SMQ > 50
Cao et al. (53)	48,870	70,158			Middle student	A	Chengdu	N	OF	DA	2.6–10	SMQ > 50
Yao et al. (54)	9	84	21	84	Undergraduate	A D	N	N	OF	DA	2.27–28	GAD-7 ≥ 5 PHQ-9 ≥ 5
Zhu et al. (55)	687	1,482	894	1,482	Undergraduate	A D	33 provinces	21 ± 3	OF	DC	1.30–2.13	GAD-7 ≥ 5 PHQ-9 ≥ 5
Zhang et al. (56)	237	1,538	528	1,486	Undergraduate	A D	Neimenggu	17–33	OF	N	N	GAD-7 ≥ 5 PHQ-9 ≥ 5
Zhang and Chang (57)			255	706	Undergraduate	D	33 provinces	N	OF	N	N	Self-design
Ji et al. (58)	140	1,013	247	1,013	Undergraduate	A D	Sichuan	19.98 ± 1.62	OF	DC	2.14–19	SAS ≥ 50 SDS ≥ 50
Zhang et al. (59)	472	1,209	472	1,209	Undergraduate	A D	Guangdong	N	OF	DA	2.1–8	GAD-7 ≥ 5 PHQ-9 ≥ 5
Wang et al. (60)			41	396	Middle student	D	Shanxi	8–18	OF	DA	February	Self-design
Wang (61)	1,781	3,178	1,781	3,178	Undergraduate	A D	Sichuan, Yunnan, Chongqing	N	OF	DA	February	SAS ≥ 50 SDS ≥ 50
Yang et al. (62)	193	1,667	257	1,667	Undergraduate	A D	Shanxi	18–28	OF	DA	2.7–9	Self-design PQEEPH ≥ 1
Zhu and Li (63)	313	838			Undergraduate	A	Wuhan	N	N	N	N	SMQ ≥ 50

*N, number reported; RO, rapid spread and outbreak; DC, diffusion containment; DA, diffusion attenuation; OF, online self-filled; SMQ, self-made questionnaire; MMHI-60, Mental Health Inventory of Middle School Students; GAD-7, 7-item measure of Generalized Anxiety Disorder; SAS, the Self-rating Anxiety Scale; HAMA, Hamilton Anxiety Scale; PQEEPH, Psychological Questionnaires for Emergent Events of Public Health; SCL-90, Self-report Inventory; PHQ-9, 9-item Patient Health Questionnaire; SCARED, the Screen for Child Anxiety Related Emotional Disorders; SDS, Self-rating Depression Scale*.

### Quality Assessment

The main features of the 31 articles are summarized in Appendix B. The AHRQ scores illustrated that most studies scored seven to nine and were considered high-quality. However, some studies did not explain missing data or clarify whether they conducted a follow-up.

### Risk of Bias

The funnel plot was asymmetric in visualization, suggesting that publication bias may have been present, as shown in Appendix C. We used Egger's regression test, which demonstrated that the overall prevalence of anxiety (*t* = 4.73, *p* > 0.05) had no publication bias. However, the overall prevalence of depression (*t* = 2.70, *p* < 0.05) was considered to have publication bias. Therefore, the trim-and-fill approach was used to examine the bias of depression which added 12 studies, the overall proportion of studies that identified depression was robust (*p* > 0.05; see Appendix D).

### Overall Prevalence of Anxiety

**Figure 2:**
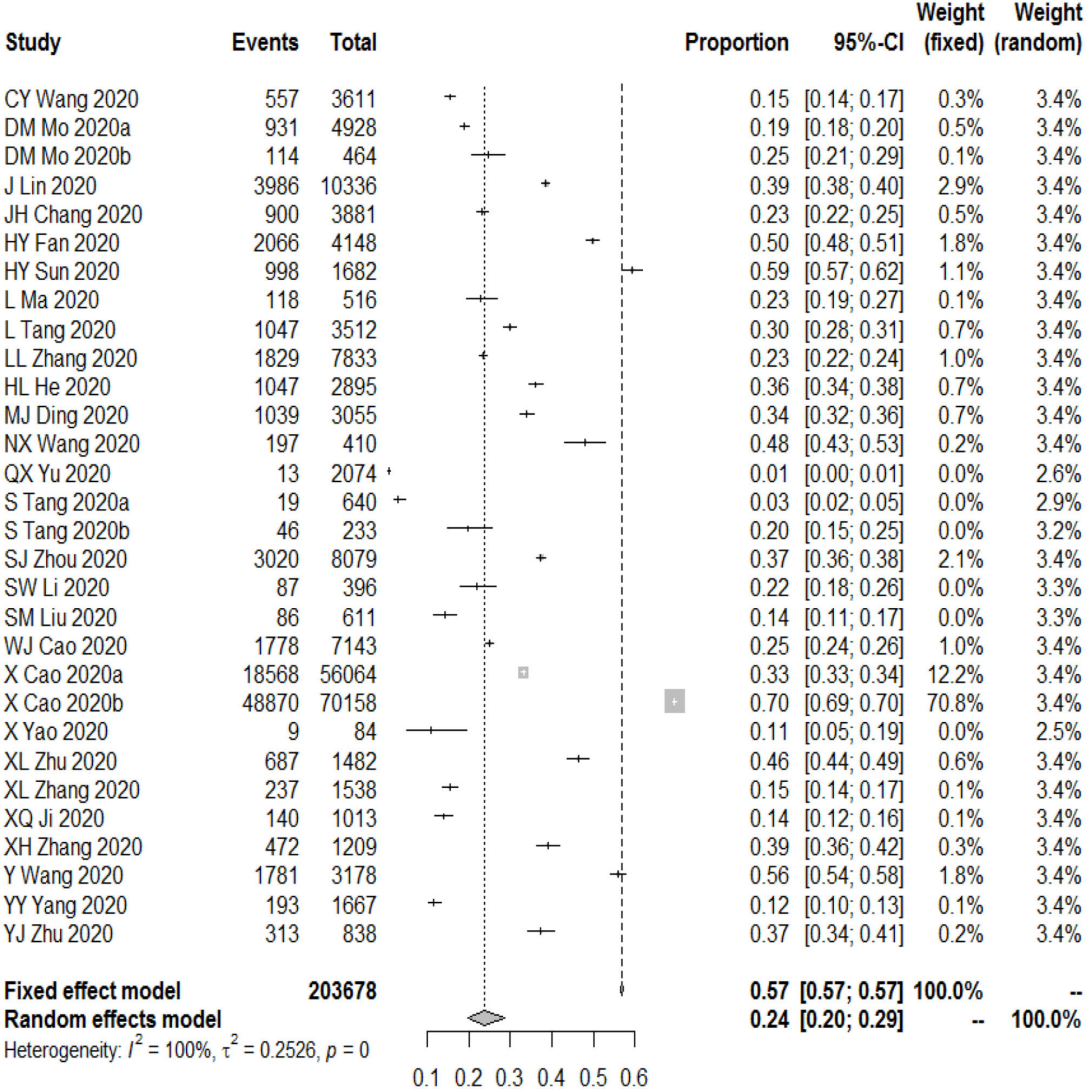
The prevalence of anxiety among Chinese students during COVID-19.

The meta-analysis showed that the overall prevalence of anxiety symptoms among Chinese students was 24.0% (95% CI [20.0–29.0%], *I*^2^ = 100%; Figure 2).

### Overall Prevalence of Depression

**Figure 3:**
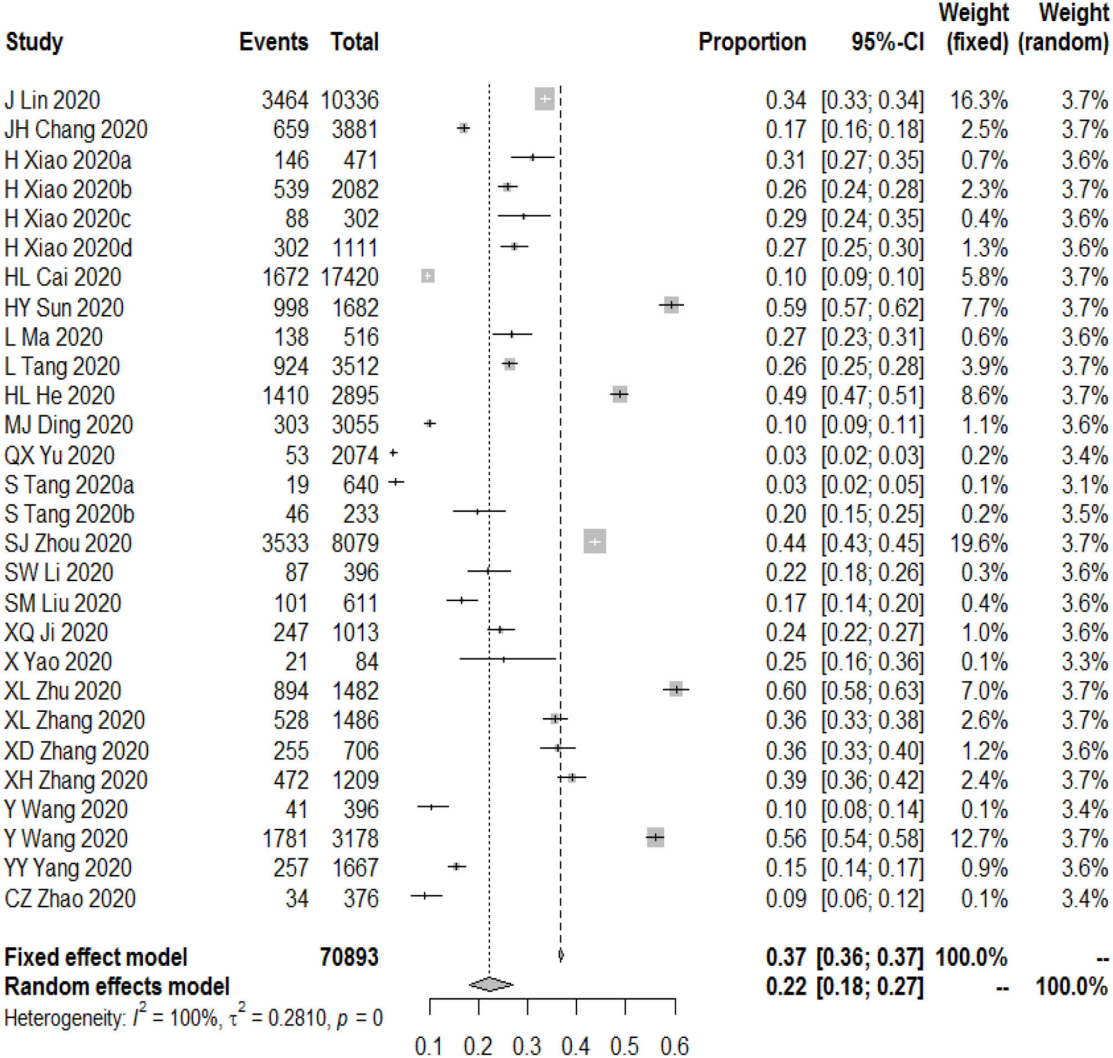
The prevalence of depression among Chinese students during the COVID-19.

The meta-analysis showed that the overall prevalence of depressive symptoms among Chinese students was 22.0% (95% CI [18.0–27.0%], *I*^2^ = 100%; Figure 3). After 12 studies were added through the trim-and-fill approach, the overall prevalence was estimated to be 40.1% (95% CI [32.9, 49.1%], *I*^2^ = 99%). This result may imply that we underestimated the prevalence of depression in Chinese students.

### Subgroup Analyses

Subgroup analyses illustrated that the prevalence of anxiety and depression was significantly moderated by pandemic area, development process, and study grade. The prevalence of anxiety (37.0%, 95% CI [34.0–41.0%]) and depression (28.0%, 95% CI [24.0–31.0%]) in the highest-risk areas of Wuhan and Hubei provinces was higher than that of other risk areas (e.g., Beijing, Guangzhou, Shanxi, Qinghai). With respect to grade, middle school students (28.0%, 95% CI [14.0–50.0%]) had a higher prevalence of anxiety than did university students (26.0%, 95% CI [19.0–34.0%]) and elementary school students (15.0%, 95% CI [6.0–33.0%]). However, the prevalence of depression in university students (27.0%, 95% CI [0.21–35.0%]) was significantly higher than that of middle (21.0%, 95% CI [10.0–39.0%]) and elementary (3.0%, 95% CI [2.0–5.0%]) school students. Additionally, the prevalence of anxiety decreased from 25.0 to 22.0% as the COVID-19 pandemic developed from rapid spread to the diffusion containment period. The prevalence of depression decreased from 25.0% in the rapid spread stage to 20.0% in the diffusion containment period. However, notably, the level of anxiety and depression increased to (42.0%, 95% CI [35.0–50.0%]) and (44.0%, 95% CI [43.0–45.0%]), respectively, in the period of diffusion attenuation. All detailed information is shown in Table 2.

**Table 2:** Subgroup analyses of anxiety and depression.

**Variable**		**Anxiety**				**Depression**			
		***k***	***I^**2**^* (%)**	**SP (%)**	**95% CI (%)**	***k***	***I^**2**^* (%)**	**SP (%)**	**95% CI**
Study grade	Primary students	4	100	0.15	[0.06, 0.33]	1	NA	0.03	[0.02, 0.05]
	Middle students	10	100	0.28	[0.14, 0.50]	7	100	0.21	[0.10, 0.39]
	University students	16	100	0.26	[0.19, 0.34]	20	100	0.27	[0.21, 0.35]
Pandemic risk areas	High-risk area	1	NA	0.37	[0.34, 0.41]	2	58	0.28	[0.24, 0.31]
	Medium-risk area	7	100	0.33	[0.21, 0.49]	4	0	0.26	[0.25, 0.27]
	Lower-risk area	3	100	0.24	[0.14, 0.37]	4	99	0.19	[0.11, 0.30]
	Low-risk area	8	100	0.17	[0.07, 0.36]	8	100	0.18	[0.09, 0.33]
Pandemic period	Occurrence and recessive spread	1	100	0.22	[0.18, 0.26]	1	NA	0.22	[0.18, 0.26]
	Rapid spread and outbreak	6	99	0.25	[0.17, 0.34]	5	100	0.25	[0.13, 0.42]
	Diffusion containment	15	100	0.22	[0.12, 0.37]	15	100	0.20	[0.13, 0.42]
	Diffusion attenuation	2	89	0.42	[0.35, 0.50]	1	NA	0.44	[0.43, 0.45]

*SP, summarized proportion; 95% CI, 95% confidence interval; NA, only one study did not have a value for the heterogeneity test*.

Analysis of the extent to which the measurement tool moderated the prevalence of anxiety and depression of Chinese students revealed that GAD-7 and self-designed questionnaires were associated with a higher prevalence of anxiety compared to SCARED and SAS. Furthermore, SDS, PHQ-9, self-designed questionnaires, and SCL-90 indicated a higher prevalence of depression compared to PQEEPH and CDI. These results imply that the overall prevalence of anxiety and depression of Chinese students was likely overestimated (Table 3).

**Table 3:** Subgroup analyses of anxiety and depression.

		***k***	***I^**2**^* (%)**	**SP (%)**	**95% CI (%)**
Anxiety	SCARED	3	68	21.0	[18.0%, 24.0%]
	SAS	7	100	19.0	[9.0%, 36.0%]
	Self-design	3	100	28.0	[13.0%, 50.0%]
	GAD-7	10	100	29.0	[21.0%, 38.0%]
	SMQ	3	100	30.0	[23.0%, 39.0%]
Depression	PHQ-9	12	100	30.0	[23.0%, 39.0%]
	SDS	3	100	30.0	[15.0%, 52.0%]
	PQEEPH	3	99	6.0	[2.0%, 21.0%]
	Self-design	7	100	26.0	[16.0%, 39.0%]
	CDI	2	96	8.0	[2.0%, 27.0%]
	SCL-90	1	NA	27.0	[23.0%, 31.0%]

*SP, summarized proportion; 95% CI, 95% confidence interval NA, only one study did not have a value for the heterogeneity test; GAD-7, 7-item measure of Generalized Anxiety Disorder; SAS, the Self-rating Anxiety Scale; PQEEPH, Psychological Questionnaires for Emergent Events of Public Health; SCL-90, Self-report Inventory; PHQ-9, 9-item Patient Health Questionnaire; SCARED, Screen for Child Anxiety Related Emotional Disorders; SDS, Self-rating Depression Scale*.

## Discussion

To the best of our knowledge, this is the first meta-analysis to provide insights into the prevalence of anxiety and depression in Chinese students during the COVID-19 pandemic. The results revealed a high level of anxiety in Chinese students during (24.0%) vs. before (17.0%) (64) the COVID-19 pandemic. The overall level of depression among Chinese students (22.0%) was similar to that before the COVID-19 pandemic (22.2%) (65). Moreover, the factors of pandemic risk area, pandemic development process, and study grade moderated the prevalence of anxiety and depression.

### Prevalence of Anxiety During the COVID-19 Pandemic

The COVID-19 pandemic and the lockdown had an immediate negative impact on the mental health of people worldwide (10, 66). We found the total prevalence of anxiety symptoms in Chinese students during the COVID-19 pandemic (24.0%) was higher than the estimated anxiety in primary school students before the pandemic (17.0%) (64). Ravens-Sieberer et al. (67) also showed that German children and adolescents experienced higher anxiety levels than before the COVID-19 pandemic (24.1 vs. 14.9%). COVID-19 was a risk factor for mental health problems in students (18). The unprecedented “home quarantine” lockdown measures likely caused students' anxiety to increase (68). One study revealed that the sudden pandemic caused 91% of students to worry about their future personal health and that of their loved ones (11), especially students who were isolated in high-risk areas. Saurabh and Ranjan (69) indicated that quarantined children and adolescents in India experienced more anxiety (61.98%) than did non-quarantined children. Moreover, students' anxiety symptoms could be related to parent–child conflicts, poor adaptation to the surrounding environment, and excessive academic pressure due to the COVID-19 pandemic (70).

### Prevalence of Depression During the COVID-19 Pandemic

The prevalence of depression (22.0%) among Chinese students during the COVID-19 pandemic might be double that of the latest global prevalence of depression among adolescents (11.3%) (71). It is noteworthy that the prevalence of depression (22.0%) during COVID-19 was slightly lower than the prevalence (22.2%) among children and adolescents in China in the previous 30 years (65), and the 23.8% prevalence of depression among Chinese university students before COVID-19 (72). A possible explanation for the lower level of depression may be that the different measurement evaluation tools affected the results. We found the SDS, the PHQ-9, self-designed questionnaires, and the SCL-90 were associated with a higher prevalence of depression, while the PQEEPH and CDI suggested a lower prevalence. However, Bueno-Notivol et al. (23) suggested that the PHQ-9 was associated with a lower prevalence. Moreover, the online questionnaires during COVID-19 were associated with statistically higher scores than were offline instruments (73).

The reported prevalence of depression among Chinese students during the COVID-19 should be considered with caution. It is possible that the COVID-19 pandemic may be more related to anxiety than depression in students. Anxiety and depression are both emotional states associated with negative affect and have a set of common (non-specific) features. People with depression often experience considerable anxiety, but anxiety does not necessarily cause depression (74). Furthermore, anxiety is related to events that have not happened yet while depression is associated with a past events (75). Oosterhoff et al. (76) indicated that adolescents who preferred to stay at home during the pandemic reported fewer depressive symptoms. The psychological reactions caused by COVID-19 may be more future-oriented than past-oriented. Therefore, COVID-19 may be related to higher levels of anxiety in Chinese students.

### Moderating Factors of Pandemic Risk Area, Development Process, and Study Grade

Compared to the other age groups, anxiety was highest in middle school students and depression was highest in university students. Concerning the former, one potential reason may be that, compared to elementary and university students, middle school students experienced more academic stress during the COVID-19 pandemic, which made them more anxious. Moghanibashi-Mansourieh (77) indicated that the switch from in-person to online learning may have reduced the learning efficiency of students in Asian countries. Furthermore, in the Chinese context, students have a strong motivation to learn, especially when completing entrance examinations (78). Middle school students are divided into junior (3 years) and senior middle schools (3 years) in the Chinese education system (79). These middle-school students who need to prepare for senior middle school and college entrance examinations experience greater academic pressure. However, online learning may have led to poor efficiency in managing online courses and thereby reduced the effectiveness of students' learning during the pandemic (12). Thus, middle-school students are more likely to have experienced anxiety during COVID-19. Concerning the latter, loss of interest or enjoyment, feelings of guilt or low self-worth, and poor sleep or appetite may increase depression in university students. Islam et al. (80) showed that compared to younger counterparts, university students typically experienced more negative consequences due to the pandemic, both academic (e.g., failure to complete scientific research experiments) and professional (e.g., unemployment). Moreover, an increase in risk factors is likely to lead to increased depression (7). These excessive risk factors may cause older students to exhibit greater depression than younger students during the COVID-19 pandemic.

The results revealed that the levels of anxiety and depression symptoms were higher in high-risk areas (e.g., Wuhan and surrounding areas) than in other areas (medium and low-risk areas). One study indicated that children in high-risk areas were more prone to fear, anxiety, and depression (81). Further, Shi et al. (82) showed that the independent factor of living in Hubei province was associated with negative mental health outcomes. People in high-risk areas (vs. low) faced a greater risk of infection and isolation, which are established risk factors with psychological impact (83). The diagnosis and mortality rates for people in high-risk areas were very high, but the health care staff and resources to treat them were very limited. These students were likely worried about being infected by other people and how long the crisis would last (84). Furthermore, isolation and control in high-risk areas were stricter than in other areas, which may have resulted in longer periods of isolation experienced by the students (12). Under prolonged lockdown, students may have experienced increased social isolation that affected their mental health.

The results showed that students' depression and anxiety symptoms gradually increased when the pandemic spread from the occurrence to the rapid spread period. This may be due to the students being overly worried about their own lives and health due to the increasing number of confirmed cases in the initial stages of the outbreak and the inadequate response from the government and hospitals (12). Subsequently, China managed to take many measures to control the outbreak (85). With the control of the COVID-19 pandemic, the prevalence of anxiety and depression among Chinese students exhibited a downward trend. This may be due to government support and restrictions—for instance, limiting public gatherings, lockdowns, and mask-wearing mandates—causing the spread of COVID-19 to ease as well as reducing the prevalence of anxiety and depression among students (86, 87). However, interestingly, we found that the level of anxiety and depression of students rebounded in the diffusion attenuation period, even exceeding the levels measured during the outbreak period of COVID-19. The rebound in the prevalence of anxiety and depression may be related to the delayed emergence and long-term persistence of psychological disorders caused by posttraumatic stress disorder (88). Like other traumatic experiences, COVID-19, as a new type of mass trauma, may have led to posttraumatic stress disorder (64). In addition, March is the normal time for Chinese students to start school. However, due to the epidemic situation, students were required to stay at home. This continuous closure and isolation may also lead to a decline in mental health (10).

### Research Strengths and Applications

Existing studies of the impact of COVID-19 on the prevalence of anxiety and depression have limitations, such as small sample sizes (6, 54, 60), use of different psychological measures (52, 53), and inclusion of a limited number of factors associated with COVID-19 (40, 89). Furthermore, previous single studies have disputed the prevalence of anxiety (42, 54) and depression (44, 60) among Chinese students during COVID-19. In this study, we synthesized the prevalence of anxiety and depression among Chinese students during the epidemic to provide data support for understanding the mental health of students worldwide during the COVID-19 pandemic. Additionally, we explored the relationship between key factors associated with the spread of the epidemic and the prevalence of anxiety and depression. We found that the COVID-19 pandemic had a differential impact on anxiety and depression among Chinese students at different stages of study. Anxiety and depressive symptoms caused by sudden stress reactions in students due to the pandemic lasted for a long time and may have a delayed rebound. Notably, governmental and medical measures to control and support the outbreak may be important protective factors in reducing students' anxiety and depression. Therefore, during public health emergencies, government, schools, and medical departments should provide targeted psychological interventions for students in different stages, populations, and periods to promote their psychological health.

### Limitations and Future Research Potential

This study had some limitations. First, the limited number of reviewed studies restricts the generalizability of the findings. Moreover, this study only investigated the prevalence of anxiety and depression among students in China. Therefore, implications concerning other cultures should be inferred with caution. Future research should focus on differences in the prevalence of anxiety and depression among persons of different cultural backgrounds. Second, it is difficult to assess the magnitude and direction of bias in the pooled prevalence estimate because the studies included in our meta-analysis had different definitions of anxiety and depression. Caution is needed when generalizing our findings. Third, although we assessed the possible source of heterogeneity through subgroup analyses, there was high heterogeneity of anxiety and depression in this study. This heterogeneity was probably caused by other factors associated with the risk of depressive symptoms that were not identified. Future studies should consider the impact of other factors on the prevalence of anxiety and depression during the COVID-19 pandemic. Fourth, some studies had a rated medium quality level. We recommend future studies should pay more attention to study quality, in particular, in the handling of missing data and reporting follow-up. Fifth, although we performed a moderation analysis of the pandemic period, participants were different among studies. In the future, longitudinal data are needed to examine the trajectory of anxiety and depressive symptoms in Chinese students in the pandemic era. Finally, the included studies provided little information on mental health services. Mental health services for students with anxiety and depression are very important for mental health planning and policymaking in the context of the COVID-19 pandemic. Future research should consider the development of mental health services for the students during the COVID-19 pandemic.

## Conclusion

In conclusion, Chinese students demonstrated a significant increase in anxiety levels as the COVID-19 pandemic progressed. Chinese middle school students were at a heightened risk of anxiety, while university students were at a heightened risk of depression during the pandemic, especially those in higher-risk areas. The government, health, and school systems should adopt a series of effective measures to alleviate anxiety and depression symptoms of students in high-risk areas. Furthermore, mental health interventions are in urgent demand for students, especially during the diffusion containment and diffusion attenuation periods of the COVID-19 pandemic.

## Data Availability Statement

The datasets presented in this study can be found in online repositories. The names of the repository/repositories and accession number(s) can be found in the article/Supplementary Materials.

## Author Contributions

YZ and XB conceived and designed the study and wrote the paper. YZ, XB, and JY performed the statistical analysis. XB and JY conducted the format and tables. YZ, XB, and HM reviewed and edited the manuscript. Moreover, all authors have approved the final manuscript for submission.

## Conflict of Interest

The authors declare that the research was conducted in the absence of any commercial or financial relationships that could be construed as a potential conflict of interest.

## Publisher's Note

All claims expressed in this article are solely those of the authors and do not necessarily represent those of their affiliated organizations, or those of the publisher, the editors and the reviewers. Any product that may be evaluated in this article, or claim that may be made by its manufacturer, is not guaranteed or endorsed by the publisher.
